# Fat and Blood

**Published:** 1884-10

**Authors:** 


**Fat and Blood.** An Essay on the Treatment of Certain Forms of Neurasthenia and Hysteria. By S. WEIR MITCHELL, M. D., Physician to the Orthopædic Hospital and Infirmary for Diseases of the Nervous System, Etc. Third edition, revised, with additions. Philadelphia: J. B. Lippincott & Co. London: 16 Southampton Street Strand. 1884.

This valuable little book advocates employment of certain methods of renewing the vitality of feeble people by a combination of entire rest and of excessive feeding, made possible by passive exercise obtained through the steady use of massage and electricity. The success of the treatment advocated by the distinguished author has been demonstrated beyond question, since the first edition of this work was published. As the author states, it now runs the risk of being employed in cases which do not need it, and by persons who are not competent, and of thus being, in a measure, brought into disrepute. The remarkable cures which the book relates would excite distrust, were it not for the high character of Weir Mitchell. The same excellent results, too, have been reported by Playfair Goodell and many others. We commend the work most heartily to our readers.

